# The Economic Burden of Subjective Cognitive Decline, Mild Cognitive Impairment and Alzheimer's Dementia: Excess Costs and Associated Clinical and Risk Factors

**DOI:** 10.1002/alz70860_100774

**Published:** 2025-12-23

**Authors:** Eva Glaeser, Ingo Kilimann, Moritz Platen, Wolfgang Hoffmann, Frederic Brosseron, Katharina Bürger, Marie Coenjaerts, Emrah Düzel, Michael Ewers, Klaus Fließbach, Ingo Frommann, Maria Gemenetzi, Wenzel Glanz, Julian Hellmann‐Regen, Enise I Incesoy, Daniel Janowitz, Frank Jessen, Oliver Peters, Josef Priller, Alfredo Ramirez, Anja Schneider, Annika Spottke, Eike Jakob Spruth, Stefan Teipel, Michael Wagner, Bernhard Michalowsky, delcode

**Affiliations:** ^1^ German Center for Neurodegenerative Diseases, Greifswald, Mecklenburg‐West Pomerania, Germany; ^2^ Deutsches Zentrum für Neurodegenerative Erkrankungen (DZNE) and Department of Psychosomatic Medicine, Rostock University Medical Center, Rostock, Germany; ^3^ Deutsches Zentrum für Neurodegenerative Erkrankungen e. V. (DZNE), site Rostock / Greifswald, Greifswald, Mecklenburg‐Vorpommern, Germany; ^4^ Deutsches Zentrum für Neurodegenerative Erkrankungen e.V. (DZNE) and Institute for Community Medicine / University of Greifswald, Greifswald, Mecklenburg Western Pomerania, Germany; ^5^ Institute for Community Medicine, University Medicine Greifswald, Greifswald, Mecklenburg‐Vorpommern, Germany; ^6^ German Center for Neurodegenerative Diseases (DZNE), Bonn, Germany; ^7^ Institute for Stroke and Dementia Research (ISD), University Hospital, LMU, Munich, Germany; ^8^ German Center for Neurodegenerative Diseases (DZNE), Munich, Germany; ^9^ German Center for Neurodegenerative Diseases, Bonn, Germany; ^10^ German Center for Neurodegenerative Diseases (DZNE), Magdeburg, Germany; ^11^ Institute of Cognitive Neurology and Dementia Research (IKND), Otto‐von‐Guericke University, Magdeburg, Sachsen Anhalt, Germany; ^12^ German Center for Neurodegenerative Diseases (DZNE), Munich, Bavaria, Germany; ^13^ Institute for Stroke and Dementia Research (ISD), LMU University Hospital, LMU Munich, Munich, Germany; ^14^ Department of Psychiatry and Psychotherapy, University of Bonn, Bonn, Germany; ^15^ German Centre for Neurodegenerative Diseases (DZNE), Bonn, Germany; ^16^ Department for Neuropsychiatry and Neurodegenerative Diseases, Bonn, Germany; ^17^ Charite, Berlin, Germany; ^18^ German Center for Neurodegenerative Diseases (DZNE), Berlin, Germany; ^19^ Charité – Universitätsmedizin Berlin, corporate member of Freie Universität Berlin and Humboldt‐Universität zu Berlin – Institute of Psychiatry and Psychotherapy, Berlin, Berlin, Germany; ^20^ Deutsches Zentrum für Neurodegenerative Erkrankungen e. V. (DZNE) Bonn, Bonn, Germany; ^21^ Excellence Cluster on Cellular Stress Responses in Aging‐Associated Diseases (CECAD), University of Cologne, Cologne, Germany; ^22^ Department of Psychiatry, Medical Faculty, University of Cologne, Cologne, Germany; ^23^ Charité – Universitätsmedizin Berlin, corporate member of Freie Universität Berlin and Humboldt‐Universität zu Berlin, Berlin, Germany; ^24^ Department of Psychiatry and Psychotherapy, Technical University of Munich, Munich, Germany; ^25^ University of Edinburgh and UK DRI, Edinburgh, United Kingdom; ^26^ Department of Psychiatry and Psychotherapy, Charité, Charitéplatz 1, Berlin, Germany; ^27^ Department of Neurodegenerative Diseases and Geriatric Psychiatry, University of Bonn Medical Center, Bonn, Germany; ^28^ Department of Psychiatry and Glenn Biggs Institute for Alzheimer's and Neurodegenerative Diseases, San Antonio, TX, USA; ^29^ Division of Neurogenetics and Molecular Psychiatry, Department of Psychiatry and Psychotherapy, University of Cologne, Medical Faculty, Cologne, Germany; ^30^ Department of Psychiatry and Psychotherapy, University Medical Center, Bonn, Germany; ^31^ Department of Neurology, University of Bonn, Bonn, Germany; ^32^ Department of Psychiatry and Psychotherapy, Charité, Berlin, Germany; ^33^ Clinic for Psychosomatic and Psychotherapeutic Medicine, University Medicine Rostock, Rostock, Germany; ^34^ German Center for Neurodegenerative Diseases (DZNE), Rostock, MV, Germany; ^35^ Department of Old Age Psychiatry and Cognitive Disorders, University Hospital Bonn and University of Bonn, Bonn, Germany

## Abstract

**Background:**

This study assessed costs and cost‐associated factors in patients with subjective cognitive decline (SCD), mild cognitive impairment (MCI) and Alzheimer's Disease (AD) dementia compared to healthy controls.

**Method:**

Clinical data, risk factors, healthcare resource use and informal care provision were assessed in the DELCODE study. Costs were calculated from payer and societal perspectives, with multivariate regression analyses identifying cost‐associated factors.

**Result:**

Societal costs were elevated by 50% for SCD (adjusted mean 7,705€ [95%CI 6,227‐9,182€)], 199% for MCI (15,328€ [6,121‐24,535€]) and 602% for AD (36,031€ [3,075‐68,986€]) compared to controls (5,129€ [3,217‐7,042€]). ApoE ε4 negative patients showed higher costs compared to positive patients. Hypertension was associated with higher costs, while lower education status and high cholesterol reduced costs.

**Conclusion:**

Healthcare costs are already significantly elevated in early subjective and objective cognitive impairment, driven by formal and informal care. The study emphasizes the importance of early interventions to reduce the economic burden and delay progression.

